# Effects of surgery on survival of elderly patients with gallbladder cancer: A propensity score matching analysis of the SEER database

**DOI:** 10.3389/fonc.2023.1083618

**Published:** 2023-03-01

**Authors:** Xiaoming Xu, Jingzhi Wang, Qilong Duan

**Affiliations:** ^1^ Department of Gastroenterology, Jining First People’s Hospital, Jining, China; ^2^ Department of Radiotherapy Oncology, The Affiliated Yancheng First Hospital of Nanjing University Medical School, The First People's Hospital of Yancheng, Yancheng, China; ^3^ Shandong Medical College, Jinan, China

**Keywords:** PSM, old age, SEER database, OS, CSS, gallbladder cancer

## Abstract

**Background:**

Surgery is the sole curative therapy for gallbladder cancer (GBC) patients. Confronting an aging society, the demand to treat elderly patients with GBC is increasing. But there are few reports on survival benefit in elderly GBC patients treated with surgery. Therefore, we designed this population-based study to assess the survival benefit of surgery in GBC patients aged 70 years or older.

**Methods:**

GBC patients aged 70 years or older were identified in the surveillance, epidemiology, and end results cancer (SEER) database from 2010 to 2017. A 1:1 propensity score matching (PSM) analysis was conducted to balance the baseline data of patients. Overall survival (OS) and cancer-specific survival (CSS) of patients were evaluated by Kaplan-Meier analysis and compared with log-rank test. Independent risk factors associated with OS and CSS were determined by univariate and multivariate Cox proportional hazard regression analyses and subgroup analysis were performed.

**Results:**

A total of 2055 GBC patients aged 70 years or older were included in our study, with 1734 patients underwent surgery. Before PSM, the age, AJCC stage, TNM stage, and chemotherapy were significantly different between the surgery and no-surgery group (all P<0.05). Patients with surgery had significantly longer OS and CSS than those without surgery (P<0.0001). After 1:1 PSM, the differences in clinicopathological characteristics were reduced (all P>0.05). Kaplan-Meier analysis also showed patients received surgery had significantly better OS and CSS (P<0.0001). Subgroup analysis further indicated that almost all subgroups received surgery had OS and CSS advantage, especially patients aged 70-84 years old. Finally, univariate and multivariate COX regression analyses showed that age, AJCC stage and T stage were independent prognostic factors for OS and CSS in patients undergoing surgery.

**Conclusion:**

Our study found that surgery significantly improved OS and CSS in GBC patients aged 70-84 years, but more prospective studies are needed to prove our findings.

## 1 Introduction

Gallbladder cancer (GBC) is a rare tumor ranking sixth among most common gastrointestinal cancer, and the most prevalent cancer of biliary tract (1, 2). The estimated number of new GBC cases was 115,949, representing 0.6% of all cancer cases in 2020 (3). It is well known that gallbladder adenocarcinoma (GBAC) is the most common pathological type of gallbladder cancer. The elderly patients account for the vast majority of patients with gallbladder cancer, previous study showed that the median age of GBC patients was 71 years (4). The prognosis of patients with gallbladder cancer deteriorates with age, the increasing incidence and mortality rates were primarily observed in men≥60 years and in women≥70 years of age (5). Surgery is the first line of treatment for gallbladder cancer patients (6). Currently, there have not been standard treatment guidelines for GBC in the elderly patients. Treatment in these patients remains a complicated issue because of the limited evidence, pre-existing disease, and adverse drug reactions, which lead to either undertreatment or overtreatment. Study demonstrated that complication rates, length of hospital stay, and intensive care unit admissions increased with patient age (7). The benefit of surgery for the old population has been discussed, but the results were contradictory (8–10). Several studies have also shown that age is a risk factor for prognosis in patients undergoing surgery for gallbladder cancer (11, 12). Thus, whether elderly patients with gallbladder cancer can benefit from surgical treatment or not is a topic worth exploring.

Therefore, in the current study, we extracted data of elderly patients with gallbladder cancer from the Surveillance, Epidemiology, and End Results (SEER) database, in order to clarify the impact of surgery on elderly GBC patients (≥70 years old).

## 2 Materials and methods

### 2.1 Patient selection

**Figure 1:**
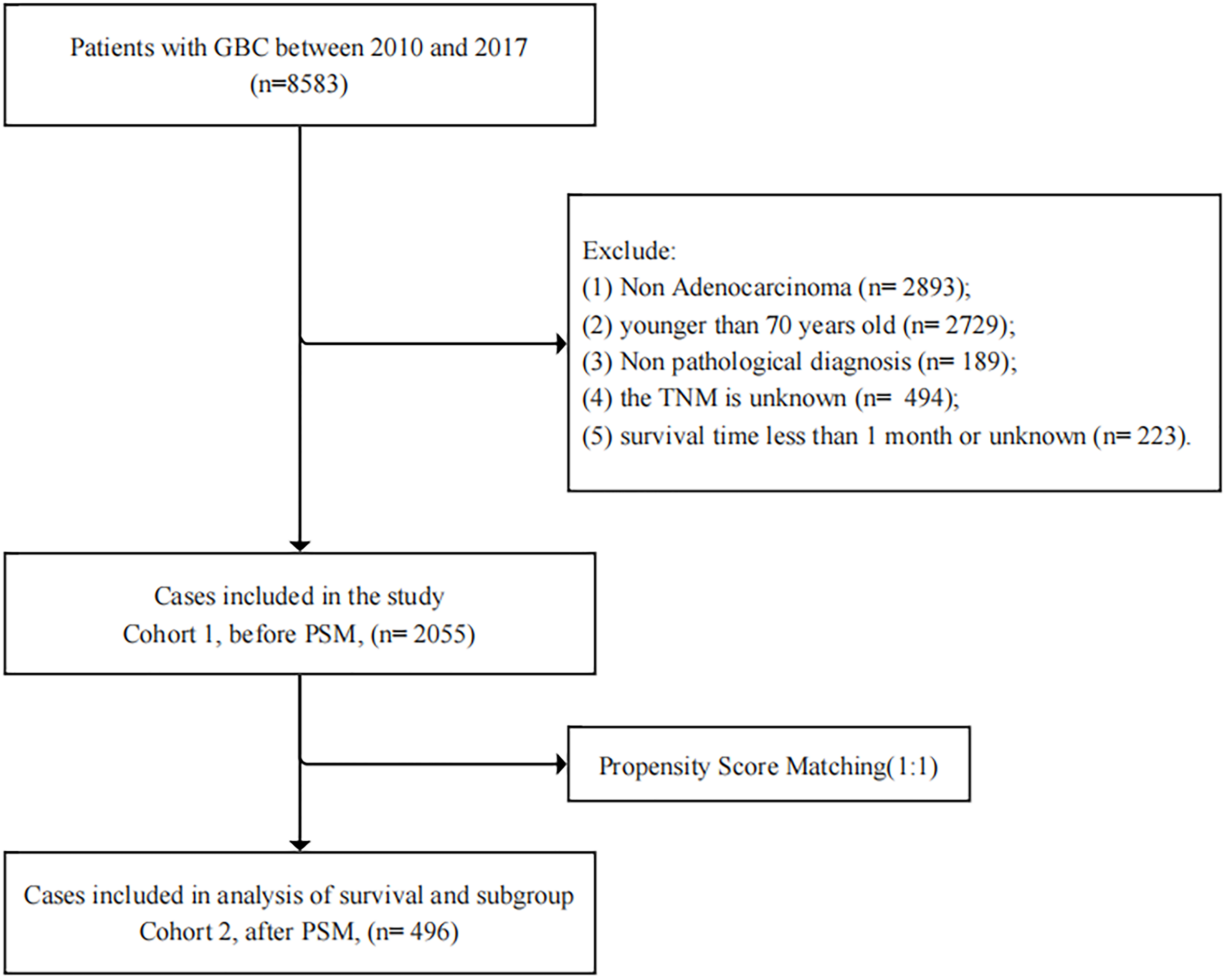
Enrollment flow chart of eligible patients in the present study.

The patient data were obtained from the SEER database, which is openly accessible and freely available for researchers. We used the SEER*Stat software with a data user agreement, the International Classification of Diseases for Oncology, 3rd Edition (ICD-O-3) Code C23.9 was used as a reference for selection. The inclusion criteria were as follows: Patients diagnosed with GBC between 2010-2017. The diagnosis was confirmed by positive histology, and the type of reporting source was not autopsy or death certificate. Patients diagnosed as non-adenocarcinoma, younger than 70 years old, survival time less than 1 month, lacking data about pathological diagnosis, TNM stage and survival were excluded. The data for patients’ sex, age, marital status, race, AJCC stage, TNM stage, surgery status, radiation status and chemotherapy status were identified. Our detailed workflow was shown in **Figure 1**.

### 2.2 Statistical analysis

Clinicopathological characteristics were compared between surgery and no-surgery group by Chi-square and Fisher’s exact probability tests. Overall survival (OS) was defined as the time from the date of diagnosis to the date of death due to any cause or the last follow-up. Cancer cause-specific survival (CSS) was defined as the time from the date of diagnosis to the date of death from cancer. Univariate and multivariate Cox proportional risk regression analyses were applied to identifying independent risk factors on survival of GBC patients. Survival analysis was accomplished by the Kaplan-Meier method and the log-rank test. Propensity score matching (PSM) was conducted to calibrate the effects of the baseline data differences. All the statistical analyses and graphics were performed with the R statistical software.

## 3 Results

### 3.1 Characteristics of patients

Among 8583 GBC patients originally identified from SEER database, cases of 1734 patients treated with surgery and 321 without surgery from 2010 to 2017 were included in our study. The clinicopathological characteristics between two groups before PSM were summarized in **Table 1**. A majority were female in both surgery and no-surgery group (67.99% vs 69.78%, P=0.571), and most of them were White (80.22% vs 76.95%, P=0.237). The proportion of patients aged 75-85 years was higher in surgery group compared with no-surgery group (72.43% vs 63.24%, P=0.005). In total, 18.4% of the patients with surgery versus 72.59% of those without surgery were AJCCIV(P<0.001). Compared with patients underwent surgery, significantly more patients in no-surgery group had TNM clinical stage of T4 (12.46% vs 1.85%, P < 0.001), N2 (9.97% vs 2.77%, P < 0.001), and M1 (64.80% vs 15.80%, P < 0.001). The PSM method was used to balance all characteristics, including sex, age, marital status, race, AJCC, TNM stage, chemotherapy, radiotherapy, and chemotherapy between surgery and no-surgery groups. And a total of 248 surgery patients were matched with 248 no-surgery patients (1:1). The clinicopathological characteristics were shown in **Table 2**. Most of the patients were female in both surgery and no-surgery group (68.15% vs 70.97%, P= 0.558). Approximately 62% patients were aged 70-79 years in both two groups (P=0.146). AJCCIVtumors (65.73% vs 64.52%, P = 0.565) as well as TNM clinical stage of T4 (7.66% vs 6.45%, P < 0.726), N2 (9.27% vs 8.06%, P < 0.213), and M1 (56.45% vs 57.26%, P=0.928) were balanced in two groups. Other characteristics, including marital status, race, radiotherapy, and chemotherapy status also showed no significantly difference between the two groups (all P>0.05).

**Table 1:** Clinical characteristics of elderly patients with GBC before propensity score matching.

Characters	No surgery (n=321)	Surgery (n=1734)	p value
Sex			0.571
Male	97 (30.22)	555 (32.01)	
Female	224 (69.78)	1179 (67.99)	
Age			0.005
70-74	118 (36.76)	478 (27.57)	
75-79	84 (26.17)	461 (26.59)	
80-84	69 (21.50)	435 (25.09)	
≥85	50 (15.58)	360 (20.76)	
Marital			0.191
No	165 (51.40)	963 (55.54)	
Married	156 (48.60)	771 (44.46)	
Race			0.237
White	247 (76.95)	1391 (80.22)	
Black	39 (12.15)	159 (9.17)	
Other	35 (10.90)	184 (10.61)	
AJCC			<0.001
I	6 (1.87)	222 (12.80)	
II	1 (0.31)	636 (36.68)	
III	81 (25.23)	557 (32.12)	
IV	233 (72.59)	319 (18.40)	
T			<0.001
T1	41 (12.77)	244 (14.07)	
T2	7 (2.18)	898 (51.79)	
T3	233 (72.59)	560 (32.30)	
T4	40 (12.46)	32 (1.85)	
N			<0.001
N0	199 (61.99)	1313 (75.72)	
N1	90 (28.04)	373 (21.51)	
N2	32 (9.97)	48 (2.77)	
M			<0.001
M0	113 (35.20)	1460 (84.20)	
M1	208 (64.80)	274 (15.80)	
Radiation			0.503
No/Unknown	282 (87.85)	1496 (86.27)	
Yes	39 (12.15)	238 (13.73)	
Chemotherapy			<0.001
No/Unknown	147 (45.79)	1262 (72.78)	
Yes	174 (54.21)	472 (27.22)	
months	4.00 (2.00, 10.00)	17.00 (6.00, 38.00)	<0.001

**Table 2:** Clinical characteristics of elderly patients with GBC after propensity score matching.

Characters	No surgery (n=248)	Surgery (n=248)	p value
Sex			0.558
Male	72 (29.03)	79 (31.85)	
Female	176 (70.97)	169 (68.15)	
Age			0.146
70-74	79 (31.85)	78 (31.45)	
75-79	75 (30.24)	76 (30.65)	
80-84	55 (22.18)	70 (28.23)	
≥85	39 (15.73)	24 (9.68)	
Marital			0.999
No	131 (52.82)	131 (52.82)	
Married	117 (47.18)	117 (47.18)	
Race			0.749
White	192 (77.42)	192 (77.42)	
Black	27 (10.89)	23 (9.27)	
Other	29 (11.69)	33 (13.31)	
AJCC			0.565
I	6 (2.42)	2 (0.81)	
II	1 (0.40)	1 (0.40)	
III	81 (32.66)	82 (33.06)	
IV	160 (64.52)	163 (65.73)	
T			0.726
T1	20 (8.06)	18 (7.26)	
T2	7 (2.82)	11 (4.44)	
T3	205 (82.66)	200 (80.65)	
T4	16 (6.45)	19 (7.66)	
N			0.213
N0	160 (64.52)	141 (56.85)	
N1	68 (27.42)	84 (33.87)	
N2	20 (8.06)	23 (9.27)	
M			0.928
M0	106 (42.74)	108 (43.55)	
M1	142 (57.26)	140 (56.45)	
Radiation			0.234
No/Unknown	220 (88.71)	210 (84.68)	
Yes	28 (11.29)	38 (15.32)	
Chemotherapy			0.928
No/Unknown	130 (52.42)	128 (51.61)	
Yes	118 (47.58)	120 (48.39)	
months	4.00 (2.00, 9.00)	8.00 (3.00, 18.00)	<0.001

### 3.2 Univariate and multivariate analysis after propensity score matching

We explored the potential independent prognosis factor for GBC patients through univariate and multivariate Cox regression analysis. Multivariate analysis showed that age≥85 years old (HR=1.661, 95%CI: 1.216-2.268, P=0.001), M1 (HR=1.774, 95%CI: 1.455-2.163, P<0.001) were significantly associated with poor OS. Surgery (HR=0.633, 95%CI: 0.527-0.761, P<0.001) and chemotherapy (HR=0.568, 95%CI: 0.466-0.694, P<0.001) were significantly associated with better OS (**Table 3**). The same results were also observed on the analysis of CSS. Age≥85 years old (HR=1.507, 95%CI: 1.076-2.111), M1 (HR=1.862, 95%CI: 1.504-2.306, P<0.001) were significantly associated with poor CSS. Surgery (HR=0.607, 95%CI: 0.498-0.739, P<0.001) and chemotherapy (HR=0.599, 95%CI: 0.484-0.741, P<0.001) were significantly associated with better CSS (**Table 4**).

**Table 3:** Univariate and multivariate Cox regression analysis of OS after propensity score matching.

	Univariate			Multivariate		
	HR	95%CI	p	HR	95%CI	p
Sex						
Male	1.000					
Female	1.018	0.836-1.238	0.862			
Age						
70-74	1.000			1.000		
75-79	1.122	0.891-1.413	0.327	0.998	0.791-1.261	0.990
80-84	0.962	0.754-1.229	0.758	0.892	0.691-1.151	0.379
≥85	2.219	1.643-2.996	<0.001	1.661	1.216-2.268	0.001
Marital						
No	1.000			1.000		
Married	0.805	0.671-0.966	0.019	0.926	0.767-1.117	0.421
Race						
White	1.000					
Black	1.044	0.770-1.416	0.779			
Other	0.958	0.730-1.257	0.757			
AJCC						
I	1.000					
II	1.070	0.222-5.163	0.933			
III	1.184	0.554-2.530	0.663			
IV	1.913	0.902-4.057	0.091			
T						
T1	1.000					
T2	0.982	0.551-1.749	0.951			
T3	1.284	0.911-1.808	0.153			
T4	1.071	0.665-1.725	0.777			
N						
N0	1.000					
N1	0.991	0.811-1.211	0.928			
N2	1.052	0.758-1.461	0.760			
M						
M0	1.000			1.000		
M1	1.699	1.408-2.048	<0.001	1.774	1.455-2.163	<0.001
Surgery						
No	1.000			1.000		
Yes	0.616	0.513-0.739	<0.001	0.633	0.527-0.761	<0.001
Radiation						
No/Unknown	1.000			1.000		
Yes	0.542	0.411-0.716	<0.001	0.791	0.590-1.061	0.118
Chemotherapy						
No/Unknown	1.000			1.000		
Yes	0.577	0.481-0.692	<0.001	0.568	0.466-0.694	<0.001

**Table 4:** Univariate and multivariate Cox regression analysis of CSS after propensity score matching.

	Univariate			Multivariate		
	HR	95%CI	p	HR	95%CI	p
Sex						
Male	1.000					
Female	1.060	0.857-1.310	0.590			
Age						
70-74	1.000			1.000		
75-79	1.081	0.844-1.384	0.539	0.970	0.755-1.245	0.808
80-84	0.966	0.745-1.253	0.794	0.905	0.69-1.187	0.469
≥85	2.036	1.471-2.817	<0.001	1.507	1.076-2.111	0.017
Marital						
No	1.000			1.000		
Married	0.733	0.602-0.892	0.002	0.837	0.684-1.023	0.083
Race						
White	1.000					
Black	1.111	0.810-1.523	0.514			
Other	0.837	0.616-1.138	0.257			
AJCC						
I	1.000					
II	0.710	0.083-6.088	0.755			
III	1.373	0.562-3.357	0.487			
IV	2.301	0.949-5.580	0.065			
T						
T1	1.000					
T2	0.968	0.513-1.827	0.920			
T3	1.347	0.926-1.958	0.119			
T4	1.042	0.616-1.762	0.877			
N						
N0	1.000					
N1	0.933	0.751-1.160	0.535			
N2	1.045	0.737-1.482	0.804			
M						
M0	1.000			1.000		
M1	1.810	1.479-2.216	<0.001	1.862	1.504-2.306	<0.001
Surgery						
No	1.000			1.000		
Yes	0.596	0.490-0.726	<0.001	0.607	0.498-0.739	<0.001
Radiation						
No/Unknown	1.000			1.000		
Yes	0.514	0.378-0.698	<0.001	0.756	0.548-1.044	0.089
Chemotherapy						
No/Unknown	1.000			1.000		
Yes	0.604	0.497-0.734	<0.001	0.599	-0.484-0.741	<0.001

### 3.3 Survival analysis of surgery and no-surgery patients

**Figure 2:**
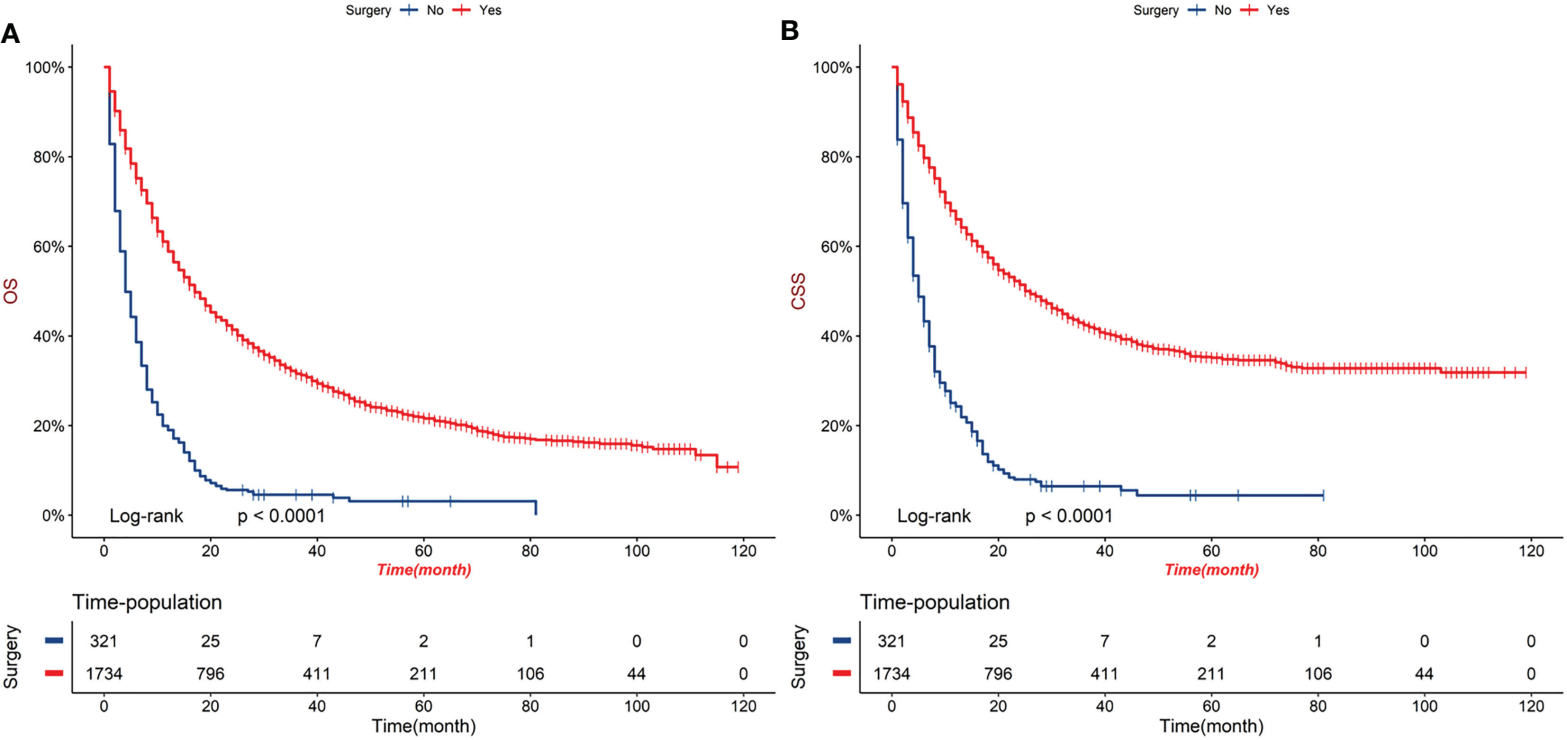
Kaplan-Meier curves of OS **(A)** and CSS **(B)** of order GBC patients before PSM.

**Figure 3:**
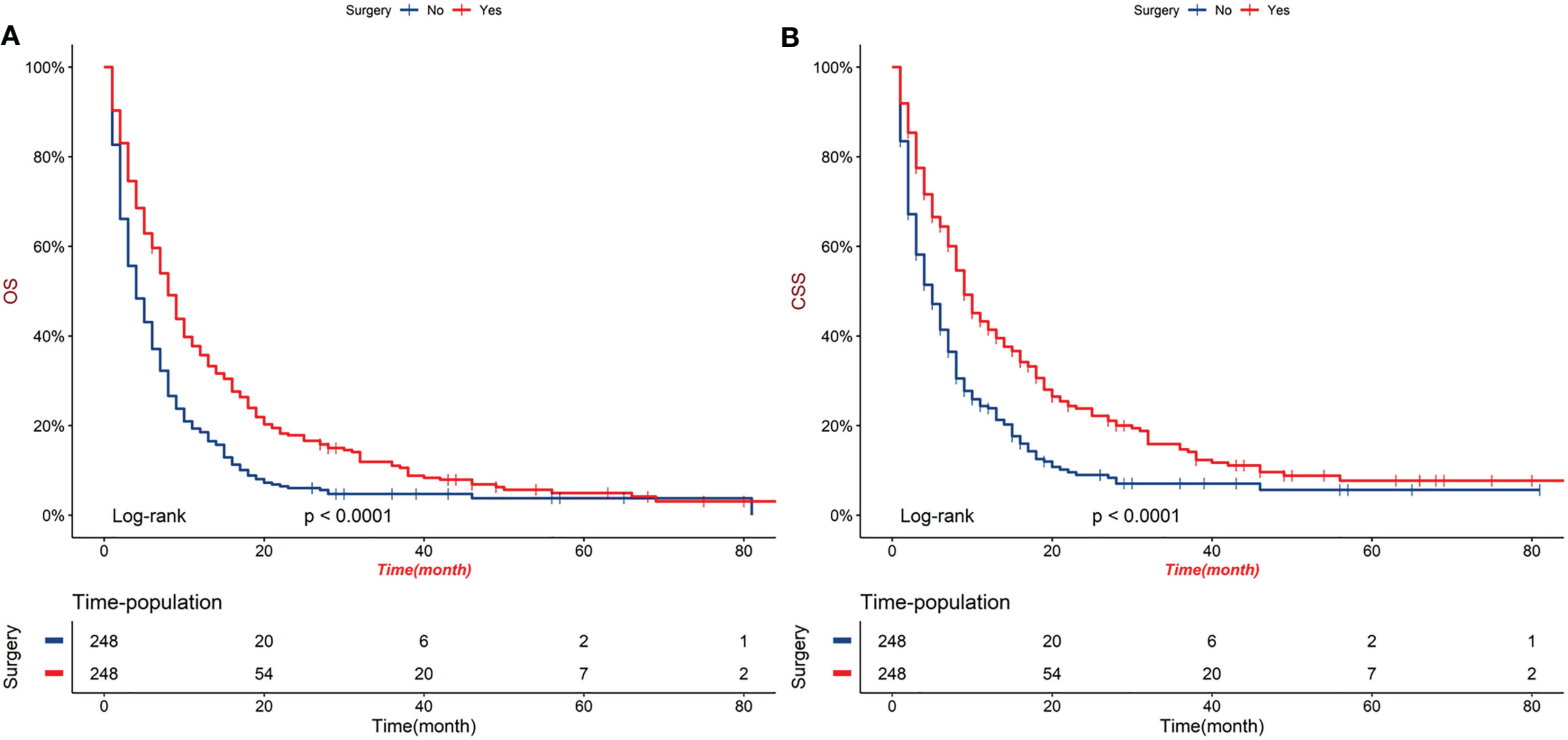
Kaplan-Meier curves of OS **(A)** and CSS **(B)** of order GBC patients after PSM.

Before PSM, patients in surgery group had significantly longer OS and CSS compared with patients in no-surgery group (median OS: 17 months vs 4 months, P < 0.001; median CSS: 26 months vs 5 months, P < 0.001, **Figure 2**). After adjusting for variables (sex, age, marital status, AJCC, TNM stage, radiotherapy and chemotherapy), surgery group still performed better OS and CSS (median OS: 8 months vs 4 months, P < 0.0001; median CSS: 9 months vs 5 months, P < 0.001, **Figure 3**).

### 3.4 Subgroup analysis of survival between surgery and no-surgery patients

**Figure 4:**
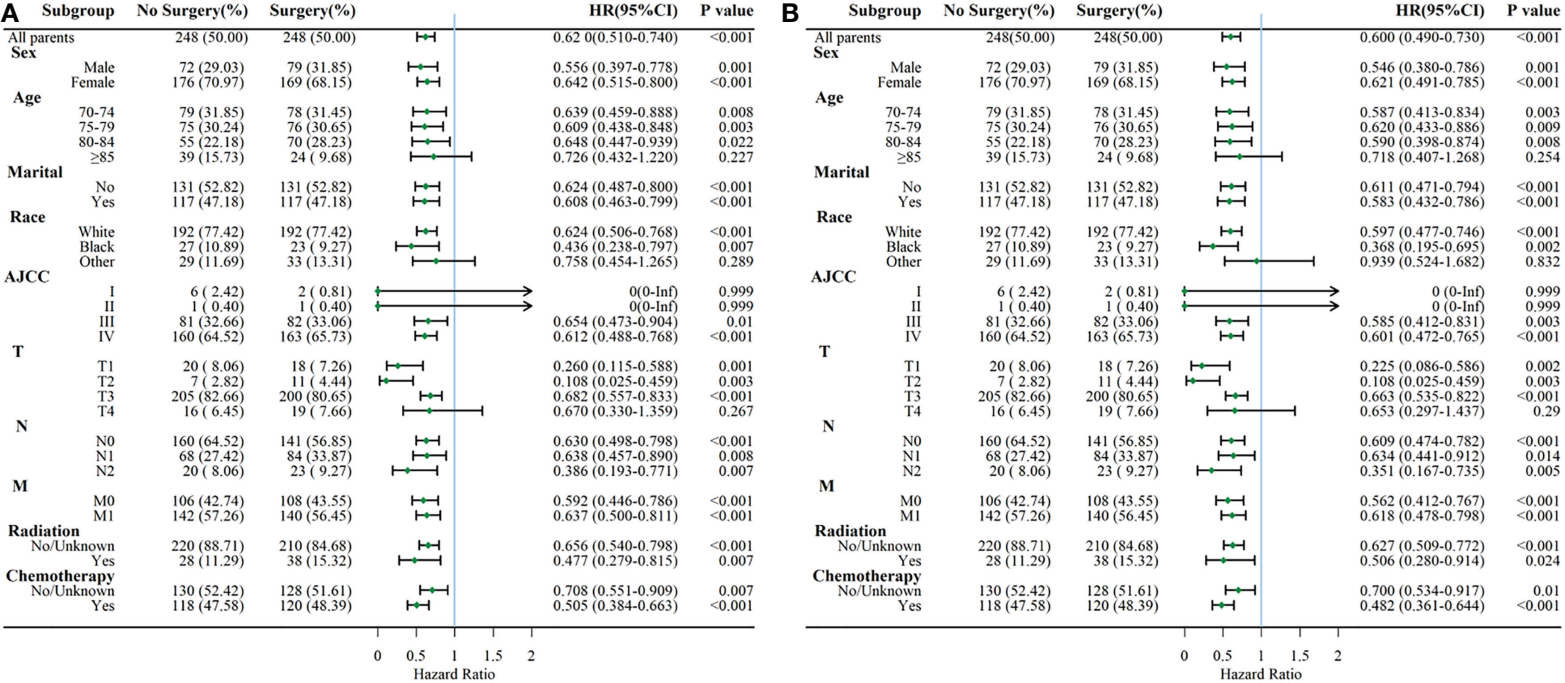
Subgroup analysis comparing OS **(A)** and CSS **(B)** between surgery and no-surgery order GBC patients.

Considering the reduction of selection bias, patients were stratified into subgroups according to the different clinical characteristics, and subgroup analysis were performed (**Figure 4**). The results showed that surgery was a protective prognostic factor for OS in almost all subgroups, including patients aged 70-84 years old. AJCCIandIIsubgroups presented insignificant differences in OS between surgery and no-surgery patients because of a small number of available cases. The CSS subgroup analysis showed that surgical treatment was a protective factor for DSS survival in the same subgroups as OS.

### 3.5 Prognostic factors of patients undergoing surgery

To further investigate the prognostic factors affecting elderly patients who underwent surgery, we performed univariate and multivariate cox regression analyses. The results showed that age, AJCC stage and T stage were independent risk factors for OS (**Table 5**) and CSS (**Table 6**). In addition, marriage was also an independent risk factor for OS but not for CSS.

**Table 5:** Univariate and multivariate Cox regression analysis of OS for patients underwent surgery.

	Univariate			Multivariate		
	HR	95%CI	p	HR	95%CI	p
Sex						
Male	1.000					
Female	1.035	0.923-1.161	0.557			
Age						
70-74	1.000			1.000		
75-79	1.187	1.021-1.381	0.025	1.253	1.077-1.458	0.004
80-84	1.315	1.131-1.528	0.000	1.412	1.212-1.646	0.000
≥85	1.621	1.386-1.896	0.000	1.963	1.668-2.311	0.000
Marital						
No	1.000			1.000		
Married	0.848	0.761-0.945	0.003	0.892	0.797-0.998	0.046
Race						
White	1.000					
Black	0.885	0.729-1.075	0.219			
Other	0.922	0.772-1.1	0.366			
AJCC						
I	1.000			1.000		
II	1.222	1.005-1.487	0.045	0.959	0.568-1.619	0.875
III	2.436	2.006-2.959	0.000	1.396	0.837-2.328	0.202
IV	4.820	3.91-5.941	0.000	2.785	1.377-5.63	0.004
T						
T1	1.000			1.000		
T2	1.338	1.12-1.6	0.001	1.279	0.787-2.079	0.321
T3	3.266	2.716-3.927	0.000	2.240	1.393-3.602	0.001
T4	3.954	2.668-5.861	0.000	1.467	0.767-2.807	0.247
N						
N0	1.000			1.000		
N1	1.404	1.236-1.596	0.000	0.973	0.828-1.145	0.744
N2	2.157	1.588-2.931	0.000	0.879	0.568-1.36	0.562
M						
M0	1.000			1.000		
M1	3.123	2.716-3.59	0.000	1.238	0.757-2.023	0.395
Radiation						
No/Unknown	1.000					
Yes	0.857	0.733-1.001	0.052			
Chemotherapy						
No/Unknown	1.000					
Yes	0.945	0.838-1.066	0.356			

**Table 6:** Univariate and multivariate Cox regression analysis of CSS for patients underwent surgery.

	Univariate			Multivariate		
	HR	95%CI	p	HR	95%CI	p
Sex						
Male	1.000					
Female	1.002	0.876-1.147	0.974			
Age						
70-74	1.000			1.000		
75-79	1.181	0.995-1.402	0.056	1.251	1.053-1.486	0.011
80-84	1.135	0.952-1.354	0.158	1.246	1.042-1.489	0.016
≥85	1.256	1.04-1.516	0.018	1.654	1.365-2.004	0.000
Marital						
No	1.000					
Married	0.910	0.801-1.034	0.148			
Race						
White	1.000					
Black	0.862	0.683-1.087	0.210			
Other	0.972	0.792-1.192	0.783			
AJCC						
I	1.000			1.000		
II	1.453	1.11-1.9	0.006	1.698	0.919-3.136	0.999
III	3.434	2.644-4.461	0.000	3.606	1.627-7.993	0.091
IV	7.583	5.776-9.956	0.000	1.475	0.834-2.607	0.002
T						
T1	1.000			1.000		
T2	1.638	1.29-2.08	0.000	1.475	0.834-2.607	0.182
T3	4.619	3.63-5.878	0.000	2.640	1.512-4.609	0.001
T4	6.209	4.008-9.62	0.000	1.956	0.942-4.062	0.072
N						
N0	1.000			1.000		
N1	1.518	1.31-1.759	0.000	0.932	0.779-1.115	0.441
N2	2.611	1.881-3.625	0.000	0.880	0.555-1.395	0.587
M						
M0	1.000			1.000		
M1	3.752	3.218-4.376	0.000	1.257	0.751-2.104	0.384
Radiation						
No/Unknown	1.000					
Yes	0.935	0.782-1.117	0.460			
Chemotherapy						
No/Unknown	1.000					
Yes	1.114	0.971-1.277	0.123			

## 4 Discussion

Surgery remains a fundamental part of GBC management and is the only potentially curative modality (13). Although many studies have reported some prognostic factors for GBC patients, including age, TNM stage, tumor size, adjuvant therapy, and pathological grade (11, 14–17), there were little data on the survival benefit of surgery in elderly patients with GBC. This special population was rarely included in randomized controlled trials that exploring the effect of surgery. Only a few observational studies have investigated this problem, but the applicability of the results was limited by the small sample sizes (18–20). Hence, we conducted this population-based study to explore the survival benefit of surgery in GBC patients aged 70 years or older.

The clinicopathological features and survival outcomes of GBC patients in surgery and no-surgery group were compared in this study. We found that patients with better AJCC and TNM stage were more likely to receive surgery (P<0.001). The AJCC and TNM stage are essential factors for judging the degree of tumor progression, choosing treatment decisions, and determining prognosis (21). GBC patients in advanced stages experienced the lowest rates of survival. Previous research demonstrated that patients with distant metastasis had higher mortality risk (HR= 2.392, 95% CI=2.027-2.823, P<.001) (14). Similarly, the present study showed that patients presented with M1 stage experienced higher mortality risk (for OS, M1 vs M0: HR=1.774; for CSS, M1 vs M0: HR=1.862, P<0.001). Subgroup analysis according to AJCC demonstrated that surgery could improve OS and CSS in elderly patients with AJCC III and IV. However, surgery did not affect OS and CSS in AJCC I andIIpatients. This might because of the relative small size of GBC patients with AJCC I andIIincluded in our study. Subgroup analysis also indicated that GBC patients aged 70 years or older with T1-3, any N and M stage could get OS and CSS benefits from surgery. Thus, the AJCC and TNM stage are helpful in selecting patients suitable for surgery and evaluating the prognosis for GBC patients. In addition, our study found that age, AJCC stage, and T stage were prognostic predictors for elderly patients with gallbladder cancer who underwent surgery, which is consistent with previous studies (16, 17, 22). This suggests that a detailed assessment of these factors is an important part of the comprehensive evaluation before receiving surgical treatment. Notably, our study also found that marriage was an independent predictor of OS in patients undergoing surgery and it was not statistically significant for CSS. Patients who are fighting cancer may benefit from the good experience and emotional support that come from marriage. These non-disease-induced interferences were corrected for in the CSS analysis.

In our study, we demonstrated that the cumulative mortality of GBC patients in surgery group was lower than that of no-surgery group, as well as after PSM. Multivariate Cox regression analysis indicated that surgery was a positive predictive factor of OS and CSS in GBC patients (for OS, HR=0.633, 95% CI=0.527-0.761, P<0.001; for CSS, HR=0.607, 95% CI=0.498-0.739, P<0.001). Subgroup analysis according to age was made in our study. Surgery significantly improved OS and CSS in patients aged 70-84 years old (P<0.05), but did not enhance survival in patients aged 85 or older (P>0.05). We assumed that increased age may account for more post-surgery complications, and the usual poorer nutritional status could decrease their resistance to complications. Considering their short remaining survival time, there will be few benefits to perform surgery in GBC patients aged ≥85 years, both patients and physicians had better not take the surgical risks. At the same time, if surgery must be performed inevitably, risk management is essential. Li P et al. showed that patients who underwent gallbladder adenocarcinoma resection older than 65 years may have a relatively poor OS (17). Xu X et al. demonstrated that GBC patients older than 70 years after surgery were also inversely correlated with survival (11). Our study provides further evidence that elderly patients aged 70-84 years with GBC can still benefit significantly from surgical treatment after a reasonable comprehensive evaluation. To our best knowledge, the present study was the first population-based study that systematically clarify the effect of surgery on patients over 70 years of age.

Our research has some strengths. First, our research was based on the SEER database, which collected clinical data from 28% of the US population. This means that our result is supported by a large amount of data. Second, compared with previous studies, our research targeted patients with GBC older than 70 years old. The present study also has some limitations. First, this was a retrospective study based on the SEER database, so selective bias was inevitable. Although we adjusted for confounding bias based on Cox regression, PSM, and subgroup analysis, these methods still failed to correct for potential unknown bias. Second, the SEER database lacks many data on factors such as basic diseases, preoperative physical status, and complications that may have a significant impact on the choice of treatment methods and prognosis of patients. More high-quality prospective studies are needed in the future to validate our conclusions.

## 5 Conclusion

In conclusion, this study demonstrated that surgery was an independent prognostic factor of OS and CSS for elderly GBC patients (≥70 years old). For patients of 70-84 years old, surgery was associated with improved OS and CSS. Future studies of prospective, randomized and multicenter trials are needed to validate our finding.

## Data availability statement

The original contributions presented in the study are included in the article/supplementary material. Further inquiries can be directed to the corresponding author.

## Ethics statement

The data of this study is obtained from the SEER database. The patients’ data is public and anonymous, so this study does not require ethical approval and informed consent.

## Author contributions

XX and QD designed the study. XX collected and analyzed the data. XX drafted the initial manuscript. QD and JW reviewed and edited the article. All authors contributed to the article and approved the submitted version.
